# Ultra-micro instrument in laparoscopic transabdominal preperitoneal (TAPP) hernioplasty

**DOI:** 10.1007/s13304-023-01715-0

**Published:** 2023-12-12

**Authors:** Xinqi Fan, Yongyong Ding, Nianfeng Sun, Yigang Chen

**Affiliations:** Wuxi No.2 People’s Hospital, Jiangnan University Medical Center, Wuxi, China

**Keywords:** Ultra-micro instrument, Minimally invasive, Laparoscopic hernia, TAPP

## Abstract

This study aimed to explore the feasibility of ultra-micro instruments in the laparoscopic repair of inguinal indirect hernia. This retrospective study included 83 patients with an indirect inguinal hernia who underwent elective surgery from January 2020 to December 2021. All patients were divided into the traditional laparoscopic group and ultra-micro laparoscopic group. The data on operation time, blood loss, ventilation time, hospital stays, complication, postoperative pain degree was collected and compared between the two groups. Of these 83 patients, 25 assigned to the ultra-micro group used ultra-micro instruments while 58 were assigned to the traditional group. The traditional group had a lower mean operation time (57.07 min) than the ultra-micro group (69.60 min)* p* < 0.05, while ultra-micro group patients had a shorter hospital stay (2 days) than the traditional group (3 days) *p* < 0.05. The ultra-micro group experienced significantly less pain for 6 h, 1 day, and 2 days postoperatively (2, 1, 0 points) compared to the traditional group (4, 2, 1 points) *p* < 0.05. There was no significant difference in blood loss, ventilation time, or complication between the two groups. Using ultra-micro instruments is safe and feasible. Patients have less postoperative pain and a smaller incision than the traditional laparoscopic instrument. It is worthy of clinical promotion.

## Introduction

Inguinal hernia repairs are the most frequently performed operations in general surgery with more than 20 million patients annually [1, 2]. Since the introduction of the laparoscopic technique into general surgery in the early 1990s [3], minimally invasive approaches to groin hernia repair have become increasingly popular and will be a more suitable alternative.

With the development of totally extraperitoneal repair (TEP) and laparoscopic transabdominal preperitoneal (TAPP) repair, minimally invasive surgical techniques have fewer postoperative complications, such as wound-related problems and early and chronic pain [4]. Therefore, finding a less invasive surgical method for hernia repair surgery is necessary.

A laparoscopic ultra-micro instrument was used to further improve this kind of operation and reduce the incision. This study aimed to compare the outcomes of inguinal hernia repair using traditional and ultra-minimally laparoscopic instruments to explore the application of ultra-minimally invasive technology in laparoscopic hernia repair.

## Materials and methods

This was a retrospective study comparing the different instruments used on 83 patients with an inguinal hernia who underwent elective surgery (TAPP) in the general surgery department, Wuxi No.2 People’s Hospital, Affiliated Wuxi Clinical College of Nantong University, from January 2020 to December 2021. Informed consent was obtained from all the patients prior to participation, and This study was performed in line with the principles of the Declaration of Helsinki. Approval was granted by the Ethics Committee of the Wuxi No.2 People's Hospital, Affiliated Wuxi Clinical College of Nantong University (accepted number: 2019Y-4). All studies were performed in accordance with relevant guidelines and regulations. These patients were divided into two groups: traditional and ultra-micro. All surgeries were performed by the same doctors.

This study included 58 traditional group and 25 ultra-micro group cases. All patients were male and had unilateral inguinal hernias. The general information of all patients was displayed in Table 1. In both groups, one case of fat liquefaction in the incision occurred in the navel hole. One case had a seroma in the traditional group while one case in the ultra-micro group had a scrotal hematoma.

**Table 1:** General data of the patients

	Traditional group (*n* = 58)	Ultra-micro group (*n* = 25)	*p* value
Sex, *n*			
Male	58	25	
Female	0	0	
Age, years [mean (SD)]	61.79 (9.35)	66.04 (12.15)	0.162
BMI, kg/m^2^ [mean (SD)]	25.07 (2.12)	24.37 (2.51)	0.282
Smoke, *n* (%)	10 (34.48)	10 (40.00)	0.682
DM, *n* (%)	7 (24.14)	8 (32.00)	0.529
Hypertension, *n* (%)	12 (41.38)	15 (60.00)	0.179

This study was based on evaluating the clinical data of patients that were maintained in the record after informed consent was obtained. The following information was collected: operation time, blood loss, pain degree, ventilation time, complications, and hospital stays. Postoperative ambulation time was not collected because patients with inguinal hernias were required to compress the operative area and reduce activity immediately after surgery.

### Surgical procedures

#### Traditional group

**Fig. 1:**
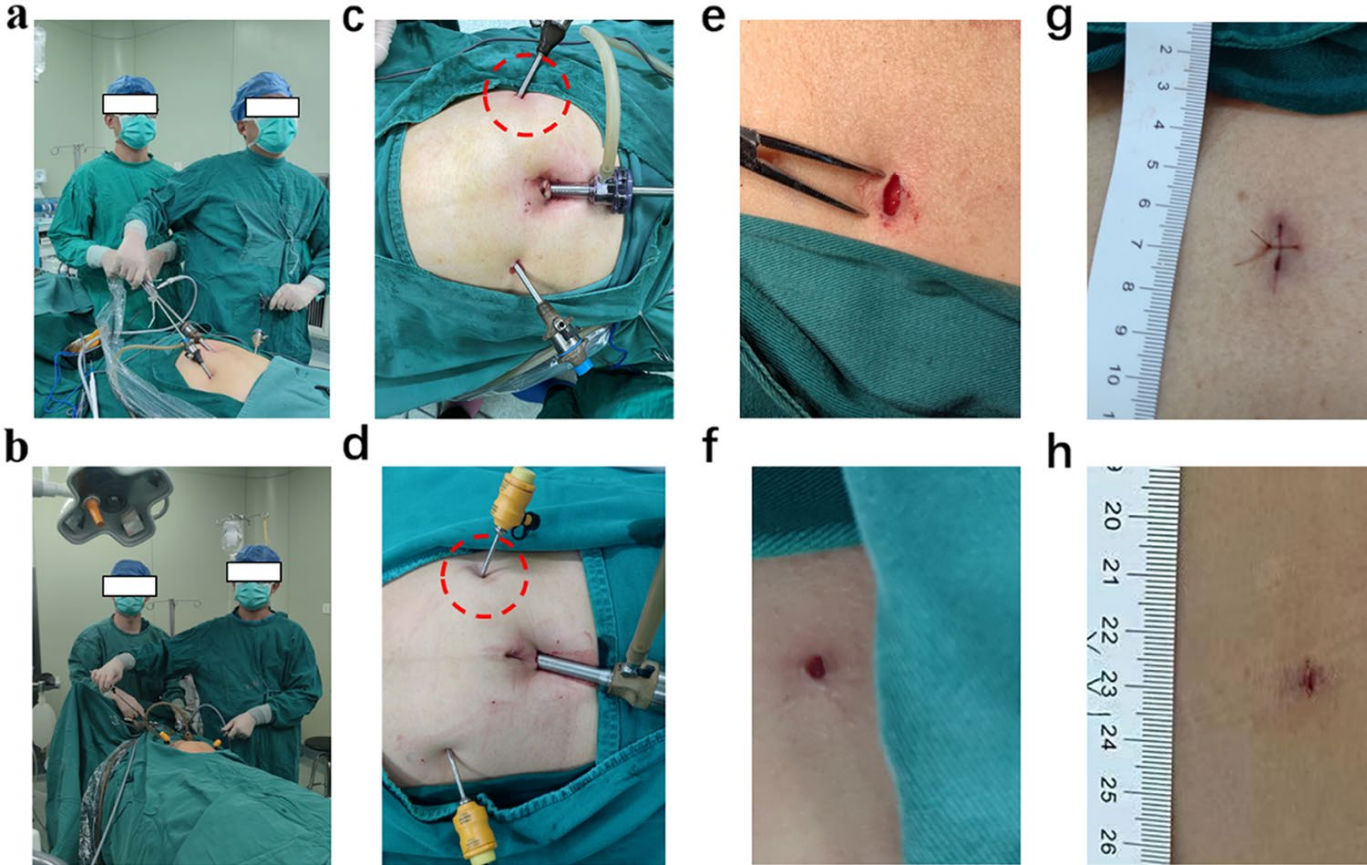
**a** Operator’s intraoperative space in the traditional group. **b** Operator’s intraoperative space in the ultra-micro group. **c** Trocar placement in the traditional group. **d** Trocar placement in the ultra-micro group. **e** Intraoperative incision in the traditional group. **f** Intraoperative incision in the ultra-micro group. **g** Postoperative incision after the suture in the traditional group. **h** Postoperative incision without suture in the ultra-micro group

A veress needle was induced pneumoperitoneum up to 12 mm Hg pressure. The 30° video camera was placed through a 10 mm trocar above the navel. The other two 5 mm trocars were put into each side of the abdomen. The operator's intraoperative space was demonstrated in Fig. 1 a, and trocar placement was demonstrated in Fig. 1c. The hernia defect was then repaired with the TAPP technique: the parietal peritoneum was incised, the hernia sac was reduced, the preperitoneal space and Cooper ligament were exposed, and funiculus’ elements or round ligament were parietalized. A polypropylene lightweight (LWM) mesh of 15–10 cm was placed in the preperitoneal space. Finally, the peritoneum was closed with a running suture. Intraoperative incision photos were revealed in Fig. 1e. The incision after the suture is shown in Fig. 1g.

#### Ultra-micro group

Gimmi laparoscopic ultra-micro instruments (Gimmi, Germany) were used in this group. The operator’s intraoperative space was demonstrated in Fig. 1b. Pneumoperitoneum was established by the same method and a 10 mm observation trocar was placed. Furthermore, the other two holes used 3 mm ultra-micro puncture devices (Fig. 1d).

**Fig. 2:**
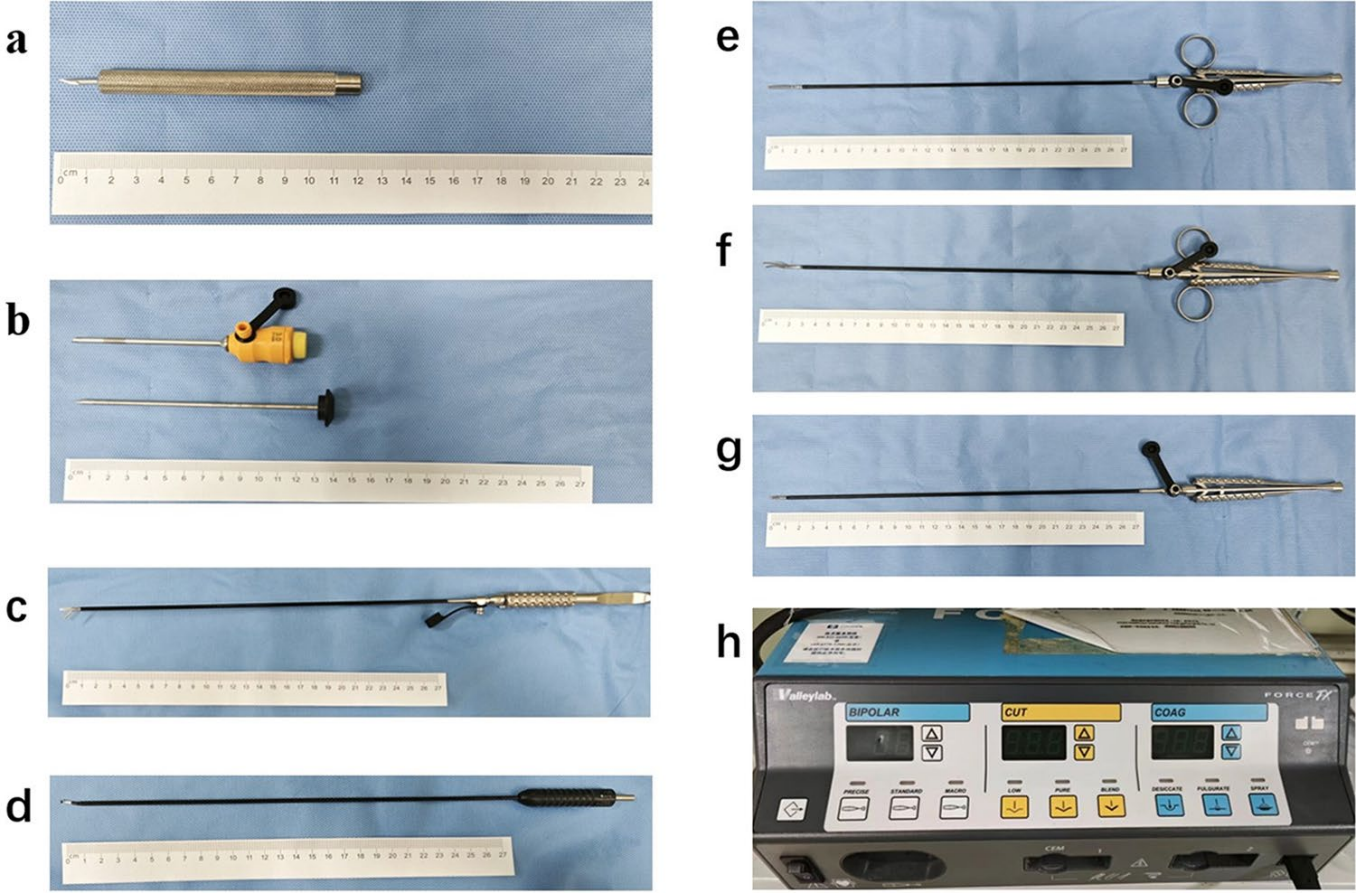
**a** Scalpel. **b** 3 mm trocar. **c** Dissection. **d** Electric hook. **e** Straight separation forceps. **f** Curved separation forceps. **g** Needle holder. **h** Energy platform

Gimmi special scalpel (Fig. 2a) was used to cut through the skin to avoid large incisions caused by conventional scalpels. 3 mm special trocars were placed. 2.7 mm ultra-micro dissection (Fig. 2c) and electric hook (Fig. 2d) were used for dissection and hemostasis. Straight separation forceps (Fig. 2e) and curved separation forceps (Fig. 2f) were used for grasping. Needle holder (Fig. 2g) was used for suturing the peritoneum. The same energy platform (Valleylab, America) was used in both groups (Fig. 2h).

The other surgery method was the same as the traditional group. Intraoperative incision photos were shown in Fig. 1f, and the incision was not sutured (Fig. 1h).

### Observed indexes

Operation time, blood loss, ventilation time, hospital stays, complications, and postoperative pain degree were collected to compare the two groups. The degree of pain was evaluated by a visual analog scale (VAS), on a scale of 0–10 points, 0 indicates no pain and 10 represents the most intense unbearable pain.

### Statistical analysis

An analysis was performed using SPSS version 26.0 software. The data subject to normal distribution were expressed as mean (SD) and analyzed by a t-test while the data not subject to normal distribution were expressed as median (range) and analyzed by the Mann–Whitney *U* test. Absolute (*n*) and relative (%) frequencies were used to describe qualitative variables analyzed by Two-way repeated analysis (two-way repeated ANOVA). The statistical significance was set at *p* < 0.05.

## Results

The traditional group’s mean operative time was 57.07 min shorter than the ultra-micro group’s 69.60 min and the difference was statistically significant (*p* < 0.05).

The mean blood loss in the traditional group was 13.10 mL compared to 14.00 mL in the ultra-micro group without a statistically significant (*p* > 0.05).

There was an insignificant difference in ventilation time between the traditional and ultra-micro groups (*p* > 0.05). The traditional group's median ventilation time was 14 h, while the ultra-micro group was 12 h.

There was a significant difference in hospital stays between the two groups (*p* < 0.05) with the median time being 3 days (traditional group) and 2 days (ultra-micro group).

Four patients had postoperative complications in 83 patients, including fat liquefaction, seroma and scrotal hematoma. There was an insignificant difference in any of the complications between the traditional and ultra-micro groups (*p* > 0.05), with one case of fat liquefaction and seroma (traditional group) compared to one case of fat liquefaction and scrotal hematoma (ultra-micro group).

Each group’s postoperative pain degree was at 6 h, 1 day, and 2 days respectively. It is important to explain median VAS points was 4 points in the traditional group contrasted with 2 points in the ultra-micro group six hours postoperatively, and 3 points contrasted with 1 point 1 day postoperatively, and 2 points contrasted 0 two days postoperatively. These differences were statistically significant (*p* < 0.05).

After follow-up, none of the patients relapsed. All data were shown in Table 2.

**Table 2:** Operative data of the patients

	Traditional group (*n* = 58)	Ultra-micro group (*n* = 25)	*p* value
Operative time, min [mean (SD)]	57.07 (5.90)	69.60 (6.91)	0.000**
Blood loss, mL [mean (SD)]	13.10 (4.31)	14.00 (5.20)	0.897
Ventilation time, h [Median (range)]	14 (10–19)	12 (9–19)	0.088
Hospital stays, d [Median (range)]	3 (2–3)	2 (2–3)	0.003**
Complication, *n* (%)			
Fat liquefaction	1 (3.45)	1 (4.00)	0.917
Seroma	1 (3.45)	0	0.358
Scrotal hematoma	0	1 (4.00)	0.286
Pain degree, point [Median (range)]			
6 h post operation	4 (3–5)	2 (2–3)	0.000**
1 day post operation	3 (2–5)	1 (1–2)	0.000**
2 days post operation	2 (1–3)	0 (0–1)	0.000**

^*^p < 0.05, **p < 0.01

## Discussions

Our research depicted insignificant differences in blood loss, ventilation time, and complications between the two groups, however, the ultra-micro group had longer operation times, shorter hospital stays, and lighter pain than the traditional group.

With the development of laparoscopic technology, laparoscopic hernia repair plays an important role in treating inguinal hernias [5, 6]. Compared to open operation, laparoscopic surgery (TAPP) has significant advantages and allows for better intraoperative exploration of the contralateral side to detect occult hernias [7–9]. However, traditional laparoscopic hernia surgery still has its drawbacks, with large trocar holes. Some scholars have suggested that by narrowing the incision, the open incision is comparable to the incision produced by traditional laparoscopy. To reduce trauma, many scholars have reduced puncture holes and developed double-hole or single-hole laparoscopic surgeries [10–13]. Currently, the feasible modified double-hole method still needs a multi-trocar operation or external assistance. However, due to the reduction of trocar holes, the operation habits changed, and the patch was difficult to place. Double pipes in the same puncture hole complicated the procedure and required considerable technology to complete the operation.

In traditional laparoscopic surgery, the skin was cut open with a scalpel, and a puncture device was inserted through the abdominal wall. The surgeon may also need to stop bleeding with an electric knife, or blunt dissection of abdominal wall tissue. A special cutting tool was used in an ultra-micro group, rather than a traditional scalpel, to precisely control the incision size, and the dilating conical trocar punch opened the remaining abdominal skin layers to retain all delicate nerves and vessels. The diameter of a traditional puncture device was 1.67 times of ultra-micro. When penetrating the abdominal wall, tissue perpendicular to the incision direction was compressed more, resulting in a wound 2.78 times the size of an ultra-micro puncture device. Due to the small incision area, no additional suturing was needed post-operative pain was reduced to a minimum.

TAPP was associated with less trauma and few postoperative complications. The time of discharge is affected by postoperative pain. Using ultra-micro devices to reduce postoperative pain can shorten hospital stays.

Furthermore, there were drawbacks of employing ultra-micro tools, such as the gripper’s small diameter and insufficient strength; it may take longer to separate and sew with finer stitches. Ultra-microinvasive instruments were similar to microscopic instruments, but different from traditional laparoscopic operation methods, resulting in a longer operation time than traditional laparoscopy. With the advancement in laparoscopic technology, local doctors doing inguinal hernia repair laparoscopically can quickly complete the learning curve by employing the same position of three trocar holes as the old method.

Our research demonstrated insignificant differences in blood loss, ventilation time, or complications between the two groups, and no patient relapsed by follow-up, suggesting that ultra-micro instruments were safe and reliable in TAPP surgery. Furthermore, there was no surgical specimen in laparoscopic hernia repair, thus there was no need to be concerned about the difficulties of specimen removal through small puncture holes. Therefore, we believe that laparoscopic hernia repair is one of the best indications for ultra-micro instruments.

The present study has several limitations. First, this study was conducted at a single institution and was retrospective in nature. Second, the number of patients was small. Further large-scale, randomized, controlled trials are needed to validate our findings.

## Conclusion

Ultra-micro instruments were safe and feasible. Patients experience less postoperative pain and a smaller incision than with traditional laparoscopic surgery, which is challenging for the surgeons’ eyes and hands. It is worthy of clinical promotion.

Our team first performed ultra-microinvasive hernia repair in Wuxi, which was highly appreciated by patients and widely reported by the media.

## References

[1] HerniaSurge Group (2018). International guidelines for groin hernia management. Hernia J Hernias Abdominal Wall Surg.

[2] Kingsnorth A, LeBlan K (2003). Herinas inguinal and incisional. Lancet.

[3] Arregui ME, Davis CJ, Yucel O (1992). Laparoscopic mesh repair of inguinal hernia using a preperitoneal approach: a preliminary report. Surg Laparosc Endosc.

[4] Aiolfi A, Cavalli M, Del Ferraro S (2021). Treatment of inguinal hernia: systematic review and updated network meta-analysis of randomized controlled trials. Ann Surg.

[5] Sains PS, Tilney HS, Purkayastha S (2006). Outcomes following laparoscopic versus open repair of incisional hernia. World J Surg.

[6] Neumayer L, Giobbie-Hurder A, Jonasson O (2004). Open mesh versus laparoscopic mesh repair of inguinal hernia. N Engl J Med.

[7] Feliu X, Claveria R, Besora P, Camps J, Fernandez-Sallent E, Vinas C, Abad JM (2011). Bilateral inguinal hernia repair: laparoscopic or open approach?. Hernia.

[8] Gainant A, Geballa R, Bouvier S, Cubertafond P, Mathonnet M (2000). Prosthetic treatment of bilateral inguinal hernias via laparoscopic approach or Stoppa procedure. Ann Chir.

[9] Krahenbuhl L, Schafer M, Schilling M, Kuzinkovas V, Buchler MW (1998). Simultaneous repair of bilateral groin hernias: open or laparoscopic approach?. Surg Laparosc Endosc.

[10] Zhou X, Song D, Miao Q, Shan W (2011). Transumbilical endoscopic surgery for completely enclosing inguinal hernias in children. Pediatr Surg.

[11] Zhou X, Peng L, Sha Y, Song D (2014). Transumbilical endoscopic surgery for incarcerated inguinal hernias in infants and children. Pediatr Surg.

[12] Jun Z, Juntao G, Shuli L, Li L (2016). A comparative study on trans-umbilical single-port laparoscopic approach versus conventional repair for incarcerated inguinal hernia in children. Minim Access Surg.

[13] Xi HW, Duan WQ, Cui QQ, You ZH, Zhao Z, Zhang P (2015). Transumbilical single-site laparoscopic inguinal hernia inversion and ligation in girls. Laparoendosc Adv Surg Tech A.

